# MEDIPIPE: an automated and comprehensive pipeline for cfMeDIP-seq data quality control and analysis

**DOI:** 10.1093/bioinformatics/btad423

**Published:** 2023-07-04

**Authors:** Yong Zeng, Wenbin Ye, Eric Y Stutheit-Zhao, Ming Han, Scott V Bratman, Trevor J Pugh, Housheng Hansen He

**Affiliations:** Princess Margaret Cancer Centre, University Health Network, Toronto, Ontario, Canada; Princess Margaret Cancer Centre, University Health Network, Toronto, Ontario, Canada; Princess Margaret Cancer Centre, University Health Network, Toronto, Ontario, Canada; Princess Margaret Cancer Centre, University Health Network, Toronto, Ontario, Canada; Princess Margaret Cancer Centre, University Health Network, Toronto, Ontario, Canada; Department of Medical Biophysics, University of Toronto, Toronto, Ontario, Canada; Princess Margaret Cancer Centre, University Health Network, Toronto, Ontario, Canada; Department of Medical Biophysics, University of Toronto, Toronto, Ontario, Canada; Ontario Institute for Cancer Research, Toronto, Ontario, Canada; Princess Margaret Cancer Centre, University Health Network, Toronto, Ontario, Canada; Department of Medical Biophysics, University of Toronto, Toronto, Ontario, Canada

## Abstract

**Summary:**

Cell-free methylated DNA immunoprecipitation and high-throughput sequencing (cfMeDIP-seq) has emerged as a promising liquid biopsy technology to detect cancers and monitor treatments. While several bioinformatics tools for DNA methylation analysis have been adapted for cfMeDIP-seq data, an end-to-end pipeline and quality control framework specifically for this data type is still lacking. Here, we present the MEDIPIPE, which provides a one-stop solution for cfMeDIP-seq data quality control, methylation quantification, and sample aggregation. The major advantages of MEDIPIPE are: (i) ease of implementation and reproducibility with Snakemake containerized execution environments that will be automatically deployed via Conda; (ii) flexibility to handle different experimental settings with a single configuration file; and (iii) computationally efficiency for large-scale cfMeDIP-seq profiling data analysis and aggregation.

**Availability and implementation:**

This pipeline is an open-source software under the MIT license and it is freely available at https://github.com/pughlab/MEDIPIPE.

## 1 Introduction

The cell-free DNA (cfDNA) in blood has been a promising analyte for cancer prognosis and treatment monitoring (Corcoran and Chabner 2018). Genomic technologies have been tailored to identify cfDNA genomic and epigenomic signatures associated with cancer phenotypes. Among them, the epigenomic profiling through cell-free methylated DNA (5-Methylcytosine) immunoprecipitation and high-throughput sequencing (cfMeDIP-seq) (Shen *et al.* 2018) has proven capable of ultrasensitive tumor detection and classification, particularly in the setting of early-stage cancer or residual disease after treatments (Shen *et al.* 2018, Nassiri *et al.* 2020, Nuzzo *et al.* 2020, Burgener *et al.* 2021, Liu *et al.* 2021, Chen *et al.* 2022, Wong *et al.* 2022, 2023). Refined cfMeDIP-seq protocols incorporating spike-in controls and/or unique molecular identifiers (UMIs) also offer improved capabilities for batch effect correction and sequence error-suppression (Shen *et al.* 2019, Burgener *et al.* 2021). The existing MeDIP-seq data analysis tools and pipelines, like MEDIPS (Lienhard *et al.* 2014), MeQA (Huang *et al.* 2012), and meDUSA (Wilson *et al.* 2012), have been adapted for cfMeDIP-seq data analysis by incorporating extra procedures, such as sequencing bias suppression, fragmental features evaluation, and spike-in controls processing. However, these studies have utilized different modified workflows, and a comprehensive, unified pipeline specifically designed for large-scale cfMeDIP-seq data processing and analysis is currently lacking. Consequently, these also challenge downstream tertiary analysis combining and comparing data across studies.

Here, we present an open-source pipeline, MEDIPIPE, to provide an automated and comprehensive solution for cfMeDIP-seq data quality control (QC), methylation quantification, and data aggregation. This pipeline was developed using Snakemake (v6.12.3) (Mölder *et al.* 2021), ensuring all dependencies are automatically installed and execution is seamless and reproducible. Through a single input configuration file, this pipeline supports various experimental settings, such as single-end or paired-end sequencing reads, and whether spike-in controls and/or UMIs were employed. Moreover, it can efficiently deal with large-scale cfMeDIP-seq profiling data on high performance computing clusters, because all independent steps for individual samples are run in parallel.

## 2 Pipeline description

**Figure 1.**
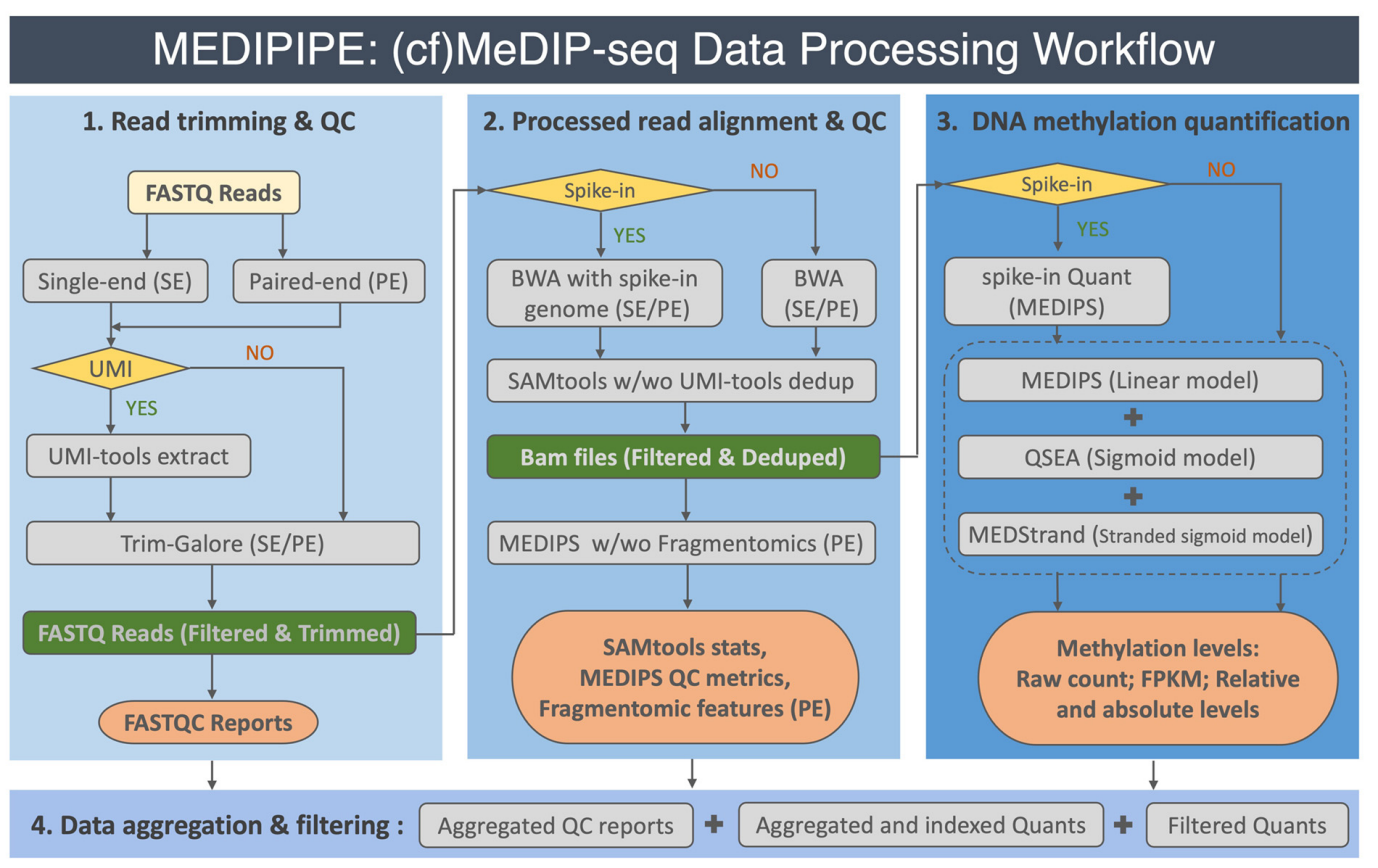
Flowchart of the MEDIPIPE pipeline. UMI, unique molecular identifiers; QC, quality control; w/wo, with or without; stats, statistics; Quant(s), quantification(s).

MEDIPIPE consists of four modules: (i) read trimming and QC; (ii) processed read alignment and QC; (iii) DNA methylation quantification; and (iv) data aggregation and filtering (Fig. 1). It starts with parsing the input configuration file with user customized experimental settings, and subsequently conducting corresponding branches in each module to get comprehensive QC and quantification outcomes (Fig. 1, Supplementary Fig. S1 and Supplementary Tables S1–S3).

### 2.1 Input of pipeline

A single input configuration file in YAML format is required for successfully running MEDIPIPE, which specifies the paths to the working environment, genome references, sample information, as well as parameters for different experimental settings (Supplementary Table S1). Instructions and a detailed template are also included in the GitHub repository. Notably, the sequencing reads can be either single-end or paired-end gzip-compressed FASTQ files, and multiple FASTQ files (e.g. multiple sequencing runs) for the same biological sample can be merged by specifying them in the sample sequencing data table (Supplementary Table S1). We also provide shell scripts for automatically downloading ENCODE pre-built genome references (e.g. BWA index and annotated regions) (Luo *et al.* 2020), or building customized genome references (e.g. when spike-in sequences need to be added to the primary genome reference).

### 2.2 Read trimming and QC

First, the adapter sequence and low low-quality bases of raw reads are removed by Trim Galore (v0.6.7) (https://www.bioinformatics.babraham.ac.uk/projects/trim_galore/). If UMI barcodes were added into the library for sequence error suppression, UMI-tools (v1.0.1) (Smith *et al.* 2017) will be executed prior to Trim Galore to extract barcodes and filter out reads which do not contain expected barcode sequence. Specifically, the regex method allowing for variable barcode length is applied. Lastly, FastQC (v0.11.9) (https://www.bioinformatics.babraham.ac.uk/projects/fastqc/) is applied to check the qualities of both raw and processed reads. All of these steps can operate on single-end or paired-end sequencing data (Fig. 1).

### 2.3 Processed read alignment and QC

Next, the processed reads are mapped back to corresponding genome(s) via BWA-MEM (v0.7.17) (Li 2013). For experiments that utilize spike-in controls, we recommend appending spike-in sequences to the primary genome for alignment simultaneously. Aligned spike-in reads can be extracted for separate quantity assessment and quantification (Wilson *et al.* 2022), which is specified in the configuration file (Supplementary Table S1). Then, SAMtools (v1.17) (Li *et al.* 2009) is applied to filter out unmapped reads and secondary alignments, as well as improperly paired mates for paired-end reads. Duplicated reads will be removed either by SAMtools markdup (Li *et al.* 2009) or UMI-tools dedup (Smith *et al.* 2017), depending on whether UMI barcodes were added or not (Fig. 1). Specific to paired-end reads, the fragment size will be estimated by CollectInsertSizeMetrics from Picard tool kit (v2.26.6) (http://broadinstitute.github.io/picard/). Fragmentation size ratios of short to long fragments in consecutive 1 and 5 Mb windows are computed as well (Cristiano *et al.* 2019). Lastly, QC metrics derived from the aligned reads, including BAM files statistics via SAMtools (Li *et al.* 2009) and cfMeDIP-seq saturation, coverage, and enrichment scores via MEDIPS (Lienhard *et al.* 2014), are collected.

### 2.4 DNA methylation quantification

The cfMeDIP-seq offers higher coverage and sensitivity of methylated CpG dinucleotides throughout the genome with lower input requirements compared to bisulfite-based methods. However, the absolute methylation levels can only be estimated using a computational model for this enrichment based profiling method. MEDIPIPE applies three successively developed methods for relative and absolute methylation estimation using different models to eliminate CpG density bias: MEDIPS (v1.46.0) (linear regression) (Lienhard *et al.* 2014), QSEA (v1.20.0) (sigmoidal model with empirical knowledge) (Lienhard *et al.* 2017) and MEDStrand (v0.0.0.9000) (stranded sigmoid model) (Xu *et al.* 2018). These options allow users to choose normalization strategy appropriate for their application and enable a comprehensive comparison among different algorithms. If there are spike-in controls, MEDIPS (Lienhard *et al.* 2014) is used to separately quantify the methylation levels for these controls as well.

### 2.5 Data aggregation and filtering

Since many cfMeDIP-seq profiling projects generate data for a large number of samples in multiple groups or batches, the final module of MEDIPIPE can aggregate QC metrics and methylation matrices across all samples for each such project. Namely, first, all QC reports generated by FASTQC, SAMtools, Picard, and MEDIPS are aggregated by MultiQC (v1.0.dev0) (Ewels *et al.* 2016) and an embedded script. On top of that, a summarized QC report in HTML format with selected QC metrics is generated, allowing users to interactively examine sample QC metrics across different groups or batches (Supplementary Fig. S2). Meanwhile, different methylation quantifications are aggregated into corresponding TAB-delimited bin-sample TXT files, as well as uniformly filtered out sex chromosomes, mitochondrial chromosome, and ENCODE blacklist regions (Amemiya *et al.* 2019). Both original and filtered aggregated quantification files are indexed by Tabix (v1.17) (Li 2011), enabling rapid retrieval of data for genomic regions of interest for downstream analysis.

### 2.6 Outputs of pipeline

All outputs of MEDIPIPE are organized in corresponding folders. Specifically, the main output files per sample can be grouped into four categories: QC reports, aligned BAM files, fragmentomic features (for paired-end reads only), and methylation quantifications (Supplementary Table S2). Moreover, the aggregated outputs include multiQC reports, aggregated QC reports, as well as the aggregated methylation quantification before and after filtering (Supplementary Table S3).

## 3 Implementation

MEDIPIPE was developed with Snakemake following a modular design in accordance with best practice coding standards so that other specialized tools can be added in the future. Detailed instructions on how to install and run this pipeline are presented in https://github.com/pughlab/MEDIPIPE. This pipeline is highly flexible thanks to the input configuration file, which provides options and parameters for different experimental settings and analyses. The pipeline can be run locally or submitted to a high performance computing system for efficient scheduling within a multiprocessor environment. As a demonstration dataset of aggregated methylation quantifications and QC reports, we have applied MEDIPIPE to the published cfMeDIP-seq dataset (Nassiri *et al.* 2020), which consists of 163 samples from 6 brain cancer subtypes (Supplementary Fig. S2). Lastly, although this pipeline was originally developed for cfMeDIP-seq data, we expect there is application for analysis of conventional methylated DNA immunoprecipitation followed by sequencing (MeDIP-seq) data.

## Supplementary Material

btad423_Supplementary_Data
