# Supplementary material for: MEDIPIPE: an automated and comprehensive pipeline for cfMeDIP-seq data quality control and analysis
Source: Bioinformatics. 2023 Jul 4;39(7):btad423. doi: 10.1093/bioinformatics/btad423 (PMC10348834; doi:10.1093/bioinformatics/btad423)
Supplement: btad423_Supplementary_Data [file btad423_supplementary_data.pdf]

Supplementary Data

MEDIPIPE: an automated and comprehensive pipeline for cfMeDIP-seq data quality control and analysis

Yong Zeng[1, #], Ye Wenbin[1], Eric Y. Stutheit-Zhao[1], Ming Han[1], Scott V. Bratman[1, 2], Trevor J. Pugh[1, 2, 3, #], Housheng Hansen He[1, 2, #]

Supplementary Figures

Supplementary Figure 1

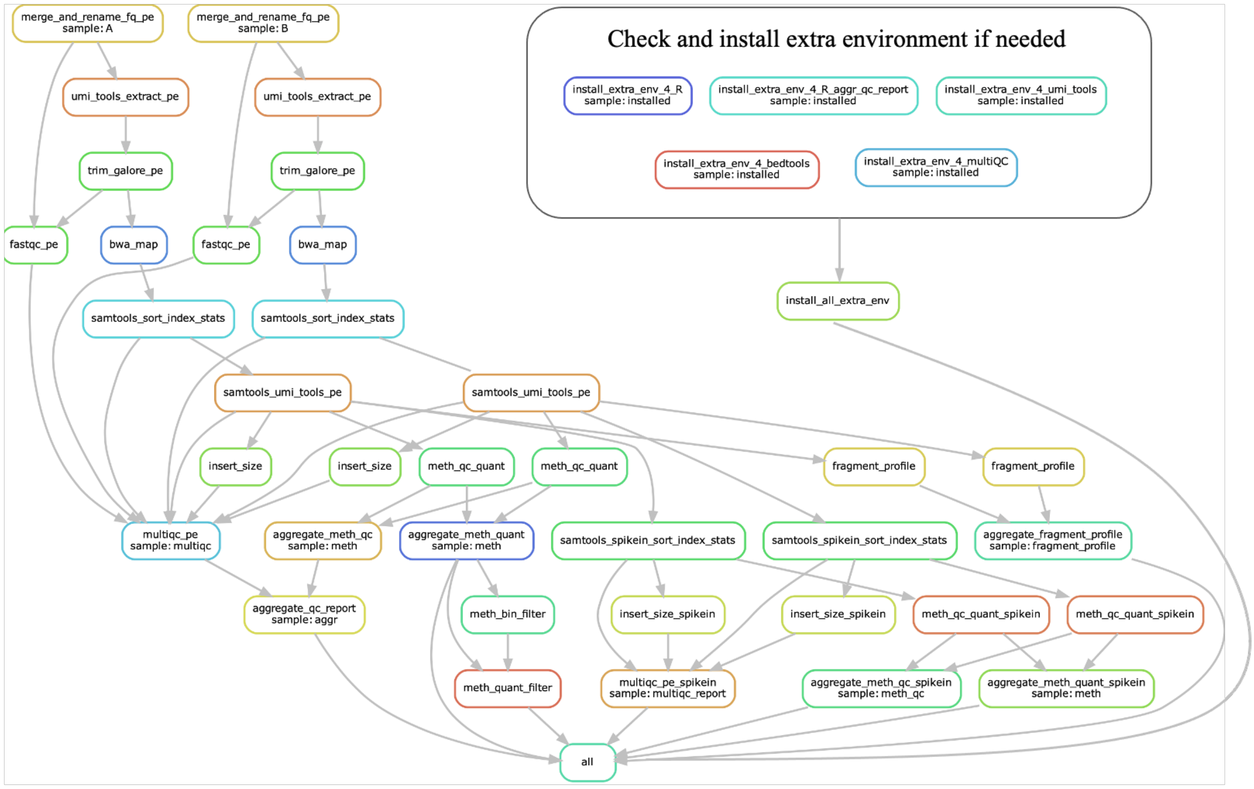

Supplementary Fig. 1. Example of detailed workflow for MEDIPIPE dealing with two paired-end cfMeDIP-seq samples (sample A and B) with UMI barcodes and spike-in controls. Each box represents the predefined Snakemake rule (specific tool and/or analysis) conducted after parsing the configuration file.

Supplementary Figure 2

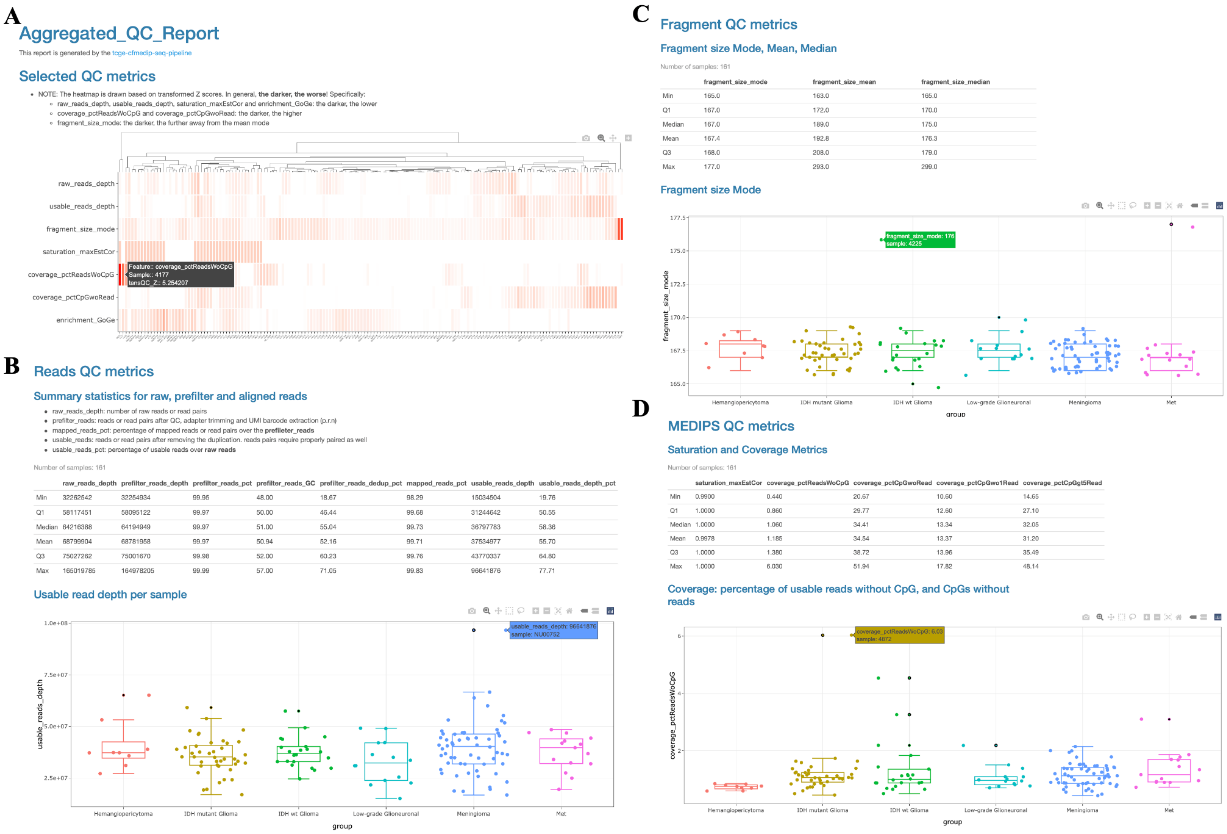

Supplementary Fig. 2. Exemplar screenshots of an aggregated, interactive QC report in HTML format. The original report (aggr_qc_report_real.html) can be downloaded from MEDIPIPE GitHub repo (./MEDIPIPE/test/Res). **(A)** Summary heatmap based on transformed Z scores of selected QC metrics. **(B, C, D)** Summary statistics for Reads, Fragment and MEDIPS QC metrics, as well as boxplots with jittered samples per groups for corresponding QC metrics.

Supplementary Tables

Supplementary Table 1. Parameters for input configuration file. Template of configuration file can also be found in pipeline GitHub repo (./MEDIPIEP/test/config_template.yaml)

Parameters	Value	Note
**PATH**		
pipe_dir	/path/to/MEDIPIPE	Full path to the MEDIPIPE clone, where the data in folder of assets and dependencies will be used for pipeline installation and running.
work_dir	/path/to/working/dir	Full path to the desired working directory.
**Samples information**		
samples	/path/to/sample_seq.tsv	Full path to the tab-separated values (TSV) file for sample sequencing information, which includes the header sample_id, R1 and R2 (as needed). Template can be found here: **./MEDIPIEP/test/sample_template.tsv**
paired-end	True or False	Indicating whether sequencing data is paired-end or not.
aggregate	True or False	Indicating whether to perform data aggregation or not.
samples_aggr	/path/to/sample_aggr.tsv<br>or NA (when aggregate == False)	Full path to the tab-separated values (TSV) file for samples to be aggregated, which includes the header sample_id and group. Template can be found here: **./MEDIPIEP/test/sample_aggr_template.tsv**
**Fragmentomic**		
frag_profile	True or False	Indicating whether to compute fragmentation size ratios of short to long fragments in 1Mb and 5Mb windows.
**Primary reference**		
ref_files	/path/to/reference.tsv	Full path to the tab-separated values (TSV) file for reference data, such as BWA index, ENCODE blacklist regions. Template can be found here: **/MEDIPIEP/test/reference_template.tsv**
bsgenome	Example:<br>BSgenome.Hsapiens.UCSC.hg38	The name of annotation R package for the primary genome. The BSgenome.Hsapiens.UCSC.hg38 and BSgenome.Hsapiens.UCSC.hg19 will be downloaded while install the pipeline, while, other annotation package needs to be downloaded priorly via specifying it in: **./MEDIPIEP/workflow/rules/extra_env/R.yaml**

**Spike-in controls**		
spike_in	True or False	Indicating whether spike-ins were added or not
spike_idx	/path/to/integrated/BWA/index<br>or NA (when spike_in == False)	Full path to the BWA index for integrated primary and spike-ins sequences. The script to build this BWA index can be found here:<br>**./MEDIPIEP/assets/Reference/build_reference_index.sh**
spike_in_bsgenome_pkg	Example for Arabidopsis BACs:<br>BSgenome.Athaliana.BAC.F19K16.F24B22_1.0.0.tar.gz<br>or NA (when spike_in == False)	The file of customized forged annotation R package for the spike-ins to be installed. More details can be found here:<br>**./MEDIPIEP/assets/README.md**
spike_in_bsgenome	Example for Arabidopsis BACs:<br>BSgenome.Athaliana.BAC.F19K16.F24B22<br>or NA (when spike_in == False)	The name of customized forged annotation R package for the spike-ins. More details can be found here:<br>**./MEDIPIEP/assets/README.md**
spike_in_chr	Example for Arabidopsis BACs:<br>AC011717.6 AL132957.1<br>or NA (when spike_in == False)	Space separated names of spike-in sequences.
**UMI barcodes**		
add_umi	True or False	Indicating whether the UMI barcodes were added or not.
umi_pattern	Example:<br>"'(?P<umi_1>^[ACGT]{3}T)'"<br>or NA (when add_umi == False)	UMI-tools regex pattern, more information about regex pattern can be found here: https://umi-tools.readthedocs.io/en/latest/regex.html#regex-regular-expression-mode
**Bin size**		
window_size	300 (by default)	The size of consecutive window size for methylation quantification for MEDIPS, QSEA and MEDStrand.

Supplementary Table 2: Output files per individual samples.

<table>
<tr><th></th><th>Files</th><th>Note</th></tr>
<tr><td rowspan="8">Quality Control Reports</td><td>./fastqc_se/Sample_ID_fastqc.html.
./fastqc_pe/Sample_ID_R1_fastqc.html
./fastqc_pe/Sample_ID_R2_fastqc.html</td><td>FASTQC for raw reads or read pairs</td></tr>
<tr><td>./barcode_fq_se/Sample_ID_extract.log
./barcode_fq_pe/Sample_ID_extract.log</td><td>UMI barcode extraction log
(if add_umi == True)</td></tr>
<tr><td>./trimmed_fq/Sample_ID.fastq.gz_trimming_report.txt
./trimmed_fq/Sample_ID_R1.fastq.gz_trimming_report.txt
./trimmed_fq/Sample_ID_R2.fastq.gz_trimming_report.txt</td><td>Automatic adapter trimming report</td></tr>
<tr><td>./fastqc_se/Sample_ID_val_1_fastqc.html
./fastqc_pe/Sample_ID_R1_val_1_fastqc.html
./fastqc_pe/Sample_ID_R2_val_1_fastqc.html</td><td>FASTQC for processed reads or read pairs</td></tr>
<tr><td>./raw_bam/Sample_ID_sorted.bam.stats.txt</td><td>Alignment statistics for unfilter BAM file</td></tr>
<tr><td>./dedup_bam_(umi)_se/Sample_ID_dedup.bam.stats.txt
./dedup_bam_(umi)_pe/Sample_ID_dedup.bam.stats.txt</td><td>Alignment statistics for deduped BAM file</td></tr>
<tr><td>./dedup_bam_spikein/Sample_ID_spikein.bam.stats.txt</td><td>Alignment statistics for deduped spike-ins BAM file (if spike_in == True)</td></tr>
<tr><td>./meth_qc_quant/Sample_ID_meth_qc.txt</td><td>MEDIPS QC : Saturation, Coverage and Enrichment Scores</td></tr>
<tr><td colspan="3"></td></tr>
<tr><td rowspan="3">Aligned Sequence</td><td>./raw_bam/Sample_ID_sorted.bam</td><td>unfilter Bam file</td></tr>
<tr><td>./dedup_bam_(umi)_se/Sample_ID_dedup.bam
./dedup_bam_(umi)_pe/Sample_ID_dedup.bam</td><td>Deduped BAM file</td></tr>
<tr><td>./dedup_bam_spikein/Sample_ID_spikein.bam</td><td>Deduped Spike-ins BAM file
(if spike_in == True)</td></tr>
<tr><td colspan="3"></td></tr>
<tr><td rowspan="3">Frangmentomic Features (For Paired-end reads only)</td><td>./fragment_size/Sample_ID_insert_size_metrics.txt</td><td>Picard fragment size estimation</td></tr>
<tr><td>./fragment_size_spikein/Sample_ID_insert_size_metrics.txt</td><td>Picard fragment size estimation for spike-ins (if spike_in == True)</td></tr>
<tr><td>./fragment_profile/Sample_ID_10_100kb_fragment_profile_GC_corrected_Ratio.txt
./fragment_profile/Sample_ID_50_100kb_fragment_profile_GC_corrected_Ratio.txt</td><td>Fragment profile (Short / Long reads ratio)</td></tr>
<tr><td colspan="3"></td></tr>
<tr><td rowspan="3">Methylation Quantification</td><td>./meth_qc_quant/Sample_ID_Granges_CpGs.bed</td><td>Bins coordinates and #CpG</td></tr>
<tr><td>./meth_qc_quant/Sample_ID_count.txt</td><td rowspan="2">MEDIPS</td></tr>
<tr><td>./meth_qc_quant/Sample_ID_rpkm.txt</td></tr>
</table>

	./meth_qc_quant/Sample_ID_rms_medips.txt	
	./meth_qc_quant/Sample_ID_rms_medestrand.txt	MEDStrand
	./meth_qc_quant/Sample_ID_nrpm_qsea.txt	QSEA
	./meth_qc_quant/Sample_ID_beta_qsea.txt	
	./meth_qc_quant/Sample_ID_logitbeta_qsea.txt	
	./meth_qc_quant/Sample_ID_CNV_qsea.txt	
	./meth_qc_quant/Sample_ID_meth_quant.RData	Combined quantifications data frame (RData) per sample

Supplementary Table 3: Output files for aggregated samples

	Files	Note
**Aggregated Quality Control Reports**	./aggregated/QC_se/multiqc_data<br>./aggregated/QC_pe/multiqc_data	MultiQC data
	./aggregated/QC_se/multiqc_report.html<br>./aggregated/QC_pe/multiqc_report.html	MultiQC HTML report
	./aggreated/meth_qc.txt	Combined MEDIPS QC metrics
	./aggreated/aggr_qc_report.csv	Aggregated selected QC metrics
	./aggreated/aggr_qc_report.html	Aggregated QC HTML report
**Aggregated methylation quantifications (Indexed with Tabix)**	./aggregated/meth_bin.bed	Bins coordinates and #CpG
	./aggregated/meth_count.txt.gz	MEDIPS
	./aggregated/meth_rpkm.txt.gz	
	./aggregated/meth_rms_medips.txt.gz	
	./aggregated/meth_rms_medestrand.txt.gz	MEDStrand
	./aggregated/meth_nrpm_qsea.txt.gz	QSEA
	./aggregated/meth_beta_qsea.txt.gz	
	./aggregated/meth_logitbeta_qsea.txt.gz	
	./aggregated/meth_CNV_qsea.txt.gz	
	./aggregated/meth_meth_quant.RData	Combined data frame per sample
**Aggregated methylation quantifications after filtering out sex chromosomes, chromosome mitochondria and ENCODE blacklist regions (Indexed with Tabix)**	./autos_bfilt/meth_autos_bfilt_bin.bed	Bins coordinates and #CpG
	./autos_bfilt/meth_count_autos_bfilt.txt.gz	MEDIPS
	./autos_bfilt/meth_rpkm_autos_bfilt.txt.gz	
	./autos_bfilt/meth_rms_medips_autos_bfilt.txt.gz	
	./autos_bfilt/meth_rms_medestrand_autos_bfilt.txt.gz	MEDStrand
	./autos_bfilt/meth_nrpm_qsea_autos_bfilt.txt.gz	QSEA
	./autos_bfilt/meth_beta_qsea_autos_bfilt.txt.gz	
	./autos_bfilt/meth_logitbeta_qsea_autos_bfilt.txt.gz	
	./autos_bfilt/meth_CNV_qsea_autos_bfilt.txt.gz	
